# Complex Haplotypes of *GSTM1* Gene Deletions Harbor Signatures of a Selective Sweep in East Asian Populations

**DOI:** 10.1534/g3.118.200462

**Published:** 2018-07-30

**Authors:** M. Saitou, Y. Satta, O. Gokcumen

**Affiliations:** * Dept. of Biological Sciences, SUNY at Buffalo, NY, 14260-1300; † Dept. of Biological Sciences, The University of Tokyo, Tokyo, Japan, 113-0033; ‡ Dept. of Evolutionary Studies of Biosystems, SOKENDAI (Graduate University for Advanced Studies), Kanagawa, Japan 240-0193

**Keywords:** copy number variants, structural variation, cellular detoxification, natural selection, soft sweep

## Abstract

The deletion of the metabolizing Glutathione S-transferase Mu 1 (*GSTM1*) gene has been associated with multiple cancers, metabolic and autoimmune disorders, as well as drug response. It is unusually common, with allele frequency reaching up to 75% in some human populations. Such high allele frequency of a derived allele with apparent impact on an otherwise conserved gene is a rare phenomenon. To investigate the evolutionary history of this locus, we analyzed 310 genomes using population genetics tools. Our analysis revealed a surprising lack of linkage disequilibrium between the deletion and the flanking single nucleotide variants in this locus. Tests that measure extended homozygosity and rapid change in allele frequency revealed signatures of an incomplete sweep in the locus. Using empirical approaches, we identified the *Tanuki* haplogroup, which carries the *GSTM1* deletion and is found in approximately 70% of East Asian chromosomes. This haplogroup has rapidly increased in frequency in East Asian populations, contributing to a high population differentiation among continental human groups. We showed that extended homozygosity and population differentiation for this haplogroup is incompatible with simulated neutral expectations in East Asian populations. In parallel, we found that the *Tanuki* haplogroup is significantly associated with the expression levels of other *GSTM* genes. Collectively, our results suggest that standing variation in this locus has likely undergone an incomplete sweep in East Asia with regulatory impact on multiple *GSTM* genes. Our study provides the necessary framework for further studies to elucidate the evolutionary reasons that maintain disease-susceptibility variants in the *GSTM1* locus.

Here, we describe the evolutionary forces that shape the variation in the *GSTM1* gene, which has been shown to be an important metabolism gene and associated with bladder cancer, autoimmune disorders and response to different drugs. Our results reveal a genetic type common in East Asian populations that may have important evolutionary and biomedical implications.

Thousands of structural variants (SVs, *i.e.*, deletions, duplications, inversions and translocations of large segments of DNA) comprise a major part of genetic variation among humans (Redon *et al.* 2006; Sudmant *et al.* 2015b). Several studies have shown that common SVs can have important functional effects, contributing to both normal and pathogenic phenotypic variation (Zhang *et al.* 2009; Weischenfeldt *et al.* 2013). In addition, there were multiple recent studies showing adaptive phenotypes that are underlain by SVs in humans (Perry *et al.* 2007; Girirajan *et al.* 2011). However, there is still a major gap in our understanding of the functional and evolutionary impact of SVs due to three interrelated factors. First, multiple mutational mechanisms lead to SV formation, affecting the size and sequence content of the resulting variants, as well as the rate of their formation (Hastings *et al.* 2009). Second, most of the functionally relevant SVs are in complex, segmental duplication regions (Feuk *et al.* 2006; Marques-Bonet *et al.* 2009). This genomic context complicates discovery and genotyping of SVs themselves and also leads to higher than usual false negative and false positive single nucleotide variation calls in those regions. Third, the segmental duplication regions also lead to frequent non-allelic homologous recombination events, leading to the formation of new SVs. Linkage disequilibrium-based imputation is essential for many evolutionary genetic analyses (Crisci *et al.* 2013) and genome-wide association studies (Visscher *et al.* 2012). Recurrent SVs and sequence exchange between homologous sequences along with recombination break the linkage disequilibrium in segmental duplication-rich loci (Usher *et al.* 2015; Boettger *et al.* 2016). As a result, the majority of SVs cannot be imputed accurately using “tag” single nucleotide polymorphisms (Wellcome Trust Case Control Consortium *et al.* 2010) and the standard, haplotype-based neutrality tests cannot be directly conducted.

To overcome these issues, recent studies have focused on resolving complex haplotype architectures harboring SVs in a locus-specific manner. For example, the reassessment of SVs in the haptoglobin locus revealed recurrent exonic deletions that are associated with blood cholesterol levels (Boettger *et al.* 2016). Similarly, the reconstruction of the haplotype-level variation in the *GYPA* and *GYPB* (Glycophorin A and Glycophorin B) locus has revealed that a specific SV leads to resistance to malaria in African populations (Leffler *et al.* 2017). To highlight some of the negative results, careful reconstruction of the haplotype-level variation in salivary *Amylase* (Usher *et al.* 2015) showed that previous associations in locus with obesity may be false. Similarly, a haplotype level analysis of salivary *MUC7* gene showed that previous association of this locus to asthma susceptibility was likely spurious but found surprising evidence of archaic hominin introgression in Africa affecting the structural variation in this locus (Xu *et al.* 2017b). Overall, with the availability of population-level genome variation datasets, it is possible to scrutinize the evolutionary and functional impact of SVs residing in complex regions of the genome.

Within this context, we focused on resolving the mechanisms and evolutionary forces that lead to the common haplotypes that carry the deletion of the glutathione S-transferase mu 1 (*GSTM1*) gene in humans (Figure 1A). *GSTM1* is a member of the *GST* gene family, which code for cellular detoxifying enzymes and studied intensively within cancer biology and pharmacogenomics context (Parl 2005). However, these enzymes are functionally relevant to human evolution as well. It has already been shown that they contribute to cellular detoxification of naturally existing products (Hayes *et al.* 2005; McIlwain *et al.* 2006). For example, GST enzymes metabolize sulforaphane, which is an abundant product in cruciferous vegetables with unknown metabolic effects (Hayes *et al.* 2005). Similarly, they metabolize prostaglandin, an important naturally existing metabolite and a target of aspirin (Ricciotti and FitzGerald 2011). Prostaglandin affects multiple evolutionarily relevant processes, including constriction of the blood vessels and platelet aggregation. As such, it is plausible that variation affecting *GST* gene family may have adaptive effects. In fact, such adaptive evolution in humans has been documented for variation affecting other metabolizing genes. These examples include but not limited to the deletion of the UDP Glucuronosyltransferase Family 2 Member B17 (*UGT2B17)* (Xue *et al.* 2008), and single nucleotide variation affecting other metabolizing genes, such as N-acetyltransferase 2 *(NAT2)* (Mortensen *et al.* 2011), alcohol dehydrogenase (Sheeley and McAllister 2008), and Glucose-6-phosphate dehydrogenase (*G6PD)* (Ruwende *et al.* 1995). Therefore, it is not a stretch to imagine that the members of the metabolizing functions of *GST* gene family are adaptively relevant. Within that context, the deletion of *GSTM1* and other variants in this locus may have a broader effect on the neighboring *GSTM* gene family, members of which have similar metabolizing functions (Hayes *et al.* 2005).

**Figure 1:**
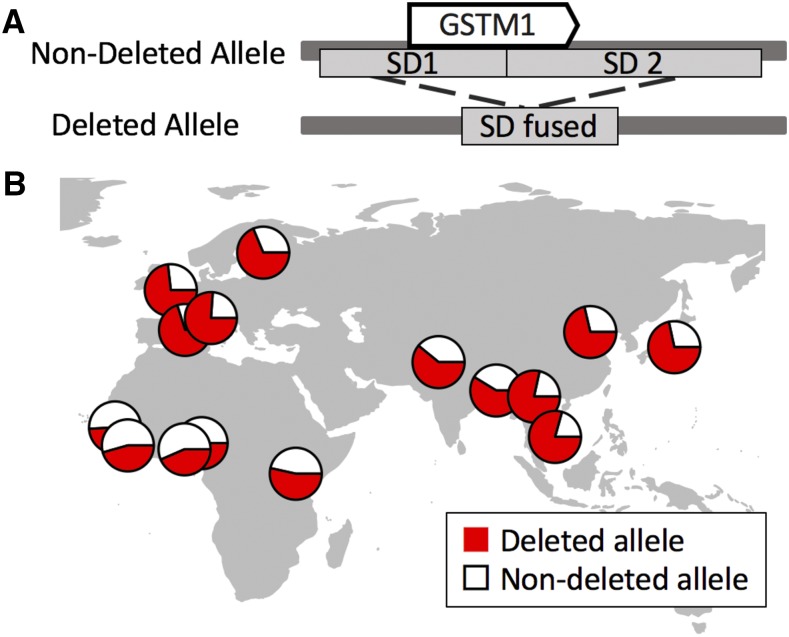
The formation of the *GSTM1* deleted allele and its geographical distribution. Two highly-similar segmental duplications fuse each other and form the deleted allele (upper). The geographical distribution of the *GSTM1* deletion allele (red) is shown in the lower figure. The frequency data are from the 1000 Genomes Project phase 3 database (Sudmant *et al.* 2015a).

In addition to its functional relevance, the *GSTM1* gene deletion is one of the most common whole gene deletions observed among human populations (Figure 1B) (Garte *et al.* 2001; Gaspar *et al.* 2002; Saadat 2007; Buchard *et al.* 2007; Fujihara *et al.* 2009; Piacentini *et al.* 2011). A whole gene deletion leads to the lack of the corresponding protein and thus it is thought to be deleterious in most cases. Therefore, it is rare for whole gene deletions to be at high frequencies (Conrad *et al.* 2010; Sudmant *et al.* 2015b). Nevertheless, the average global frequency of Eurasians with homozygous *GSTM1* deletion is higher than 50% (Sudmant *et al.* 2015a), which puts it among the most frequent whole gene deletions observed in Eurasians according to 1000 Genomes Project database, the other being deletions affecting the *LCE3BC* (Late Cornified Envelope 3B and C) and the *UGT2B17* (uridine diphosphoglucuronosyltransferase), both of which have been shown to be evolving under non-neutral conditions (Xue *et al.* 2008; Pajic *et al.* 2016).

Despite the functional and potential evolutionary importance of the *GSTM* gene family, a clear understanding of the mechanism through which the *GSTM1* deletion and other variations in this locus actually affect phenotype remains elusive. We believe that understanding the underlying evolutionary forces that shape the variation in this locus will give us a framework to better target the functionally relevant haplotypes. Such an evolutionary analysis has been difficult since the *GSTM1* gene resides in a complex genomic region with two segmental duplications (SDs) flanking both sides of the gene (Figure 1A). This particular genomic architecture predisposes *GSTM1* to non-allelic homologous recombination events, which can lead to formation of new SVs. In fact, the polymorphic deletion observed in humans was almost certainly a result of such a mechanism (Xu *et al.* 1998). Moreover, we recently reported that recurrent deletions of the *GSTM1* gene have happened independently in the human and chimpanzee lineages and that multiple rearrangements have defined genetic variation in this locus in primates (Saitou *et al.* 2018). These complicate the phylogenetic and broader evolutionary analysis immensely. This complication may also explain the paucity of evolutionary analysis in the *GSTM1* locus, which is otherwise the target of more than 500 publications in the last 20 years, most of which are single-locus association studies implicating variation in this locus in susceptibility to multiple cancers (Parl 2005).

Collectively, the functional relevance of the *GSTM1* deletion, its mechanistic complexity, and its high allele frequency in human populations make it suitable as a model for studying the evolution of metabolizing gene families. Therefore, in this paper, we analyzed hundreds of genomes to resolve the haplotypic variation that defines the *GSTM1* deletion.

## Materials and Methods

### Materials and Methods

#### Study Populations:

We primarily used data from 3 populations, YRI (Yoruba in Ibadan), CEU (Utah residents with Northern and Western European ancestry), and CHB (Han Chinese in Beijing) available from the 1000 Genome Project (1000 Genomes Project Consortium *et al.* 2012; Sudmant *et al.* 2015b). For the phylogenetic analysis, we also analyzed haplotypes of the chimpanzee reference genome (The Chimpanzee Sequencing Consortium 2005), the Neanderthal genome (Prüfer *et al.* 2014) and Denisovan genome (Reich *et al.* 2010).

#### Confirmation of the genotyping and phasing in our dataset:

To validate the accuracy of the genotyping of the *GSTM1* deletion in 1KGP dataset, we used digital droplet polymerase chain reaction experiments to genotype the *GSTM1* deletion in 17 humans from 1KGP (samples Available through Coriell Institute for Medical Research) (forward primer: TCGAGGGTGCCATTACATTC; reverse ACTTCTGTCCTGGGTCATTC; probe: /56-FAM/TAGGAGCAG /ZEN/GCAGGTGATGTGAAC /3IABkFQ/). We followed standard protocol provided by Bio-Rad EIF2C1 probe assay (Table S1).

To investigate the accuracy of the 1KGP genotyping of single nucleotide variants, we investigated 74 CHB individuals and 6 sites in the target regions (chr1:110211681-110223007 and chr1:110246810-110255596) reported both in 1KGP and HapMap (this is all the SNPs genotyped in HapMap in the region). Of all the 444 sites, 9 were “NN” in hapmap and 3 showed different results in the two databases. Overall, we found approximately %99.3 concordance between the two datasets.

To validate the accuracy of the phasing in 1KGP project data that we used in this study, we compared haplotypes on 50kb on each side of the deletion in our dataset with phased HapMap genomes. Specifically, we were able to find 322 heterozygous sites in 30 phased HapMap CEU genomes. 320 (∼99.4%) of these are concordant with the 1KGP dataset. We also investigated Sanger sequencing data from seven deleted haplotypes for which we generated long-range PCR amplicons for an earlier study (Saitou *et al.* 2018). Since, the samples that were used for Sanger sequencing were not present in 1000 Genomes database, we asked whether common polymorphic sites in 1000 Genomes are also polymorphic in the sequenced haplotypes. Here, we defined common by 30% or higher allele frequency. For the 15 common single nucleotide variants that were reported in the 1000 Genomes project that overlap with the region (**chr1:**110216300-110225620 and **chr1:**110244324-110246143, the deletion was excluded), we found 14 variants among the 7 haplotypes (one example is represented in Figure S1). The only one that was not polymorphic among the seven haplotype dataset was rs61799140. This SNP has been detected by more than a dozen previous studies based on the dbSNP (https://www.ncbi.nlm.nih.gov/projects/SNP/snp_ref.cgi?rs=61799140). As such, we concluded that the absence of polymorphism in this location among the sequenced dataset is likely because it was not represented among the seven haplotypes, rather than it being a false-positive genotype in the 1000 Genomes dataset. Overall, we are confident that 1000 Genomes adequately captures the genetic variation in this locus and that the variant calling which our further analysis based on was accurate.

#### Linkage disequilibrium and haplotype analyses:

**Figure 2:**
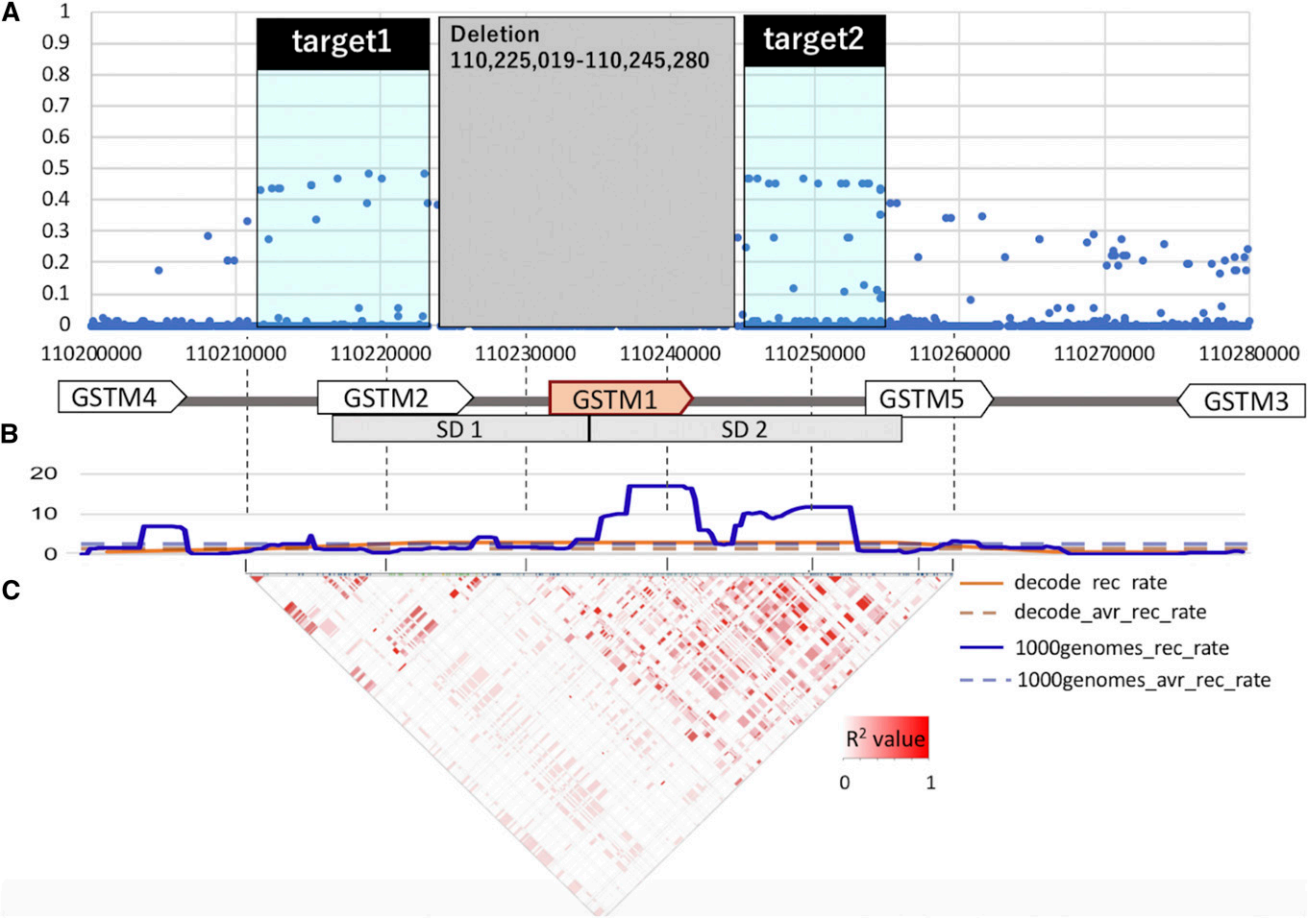
Linkage disequilibrium in the *GSTM1* region in CHB. (A) R^2^ value between the *GSTM1* deletion and flanking single nucleotide variants in CHB. Each dot represent each single nucleotide variants. X-axis indicates chromosomal location and Y-axis indicates the R^2^ value between the *GSTM1* deletion and flanking single nucleotide variants. Target regions were chr1:110211681-110223007 (target1, upstream the deletion) and chr1:110246810-110255596 (target2, downstream the deletion). (B) Recombination rate of the *GSTM1* flanking region from DeCode (orange) and 1000genomes (blue). The chromosomal average of recombination rate from decode (orange) and 1000genomes (blue) were also plotted in the graph by broken lines. (C) Linkage disequilibrium (LD, R^2^ value) between the single nucleotide variants around the *GSTM1* gene in CHB. Color coded in a continuous R^2^ values between each single nucleotide variants from 0 (white) to 1 (red).

**Figure 3:**
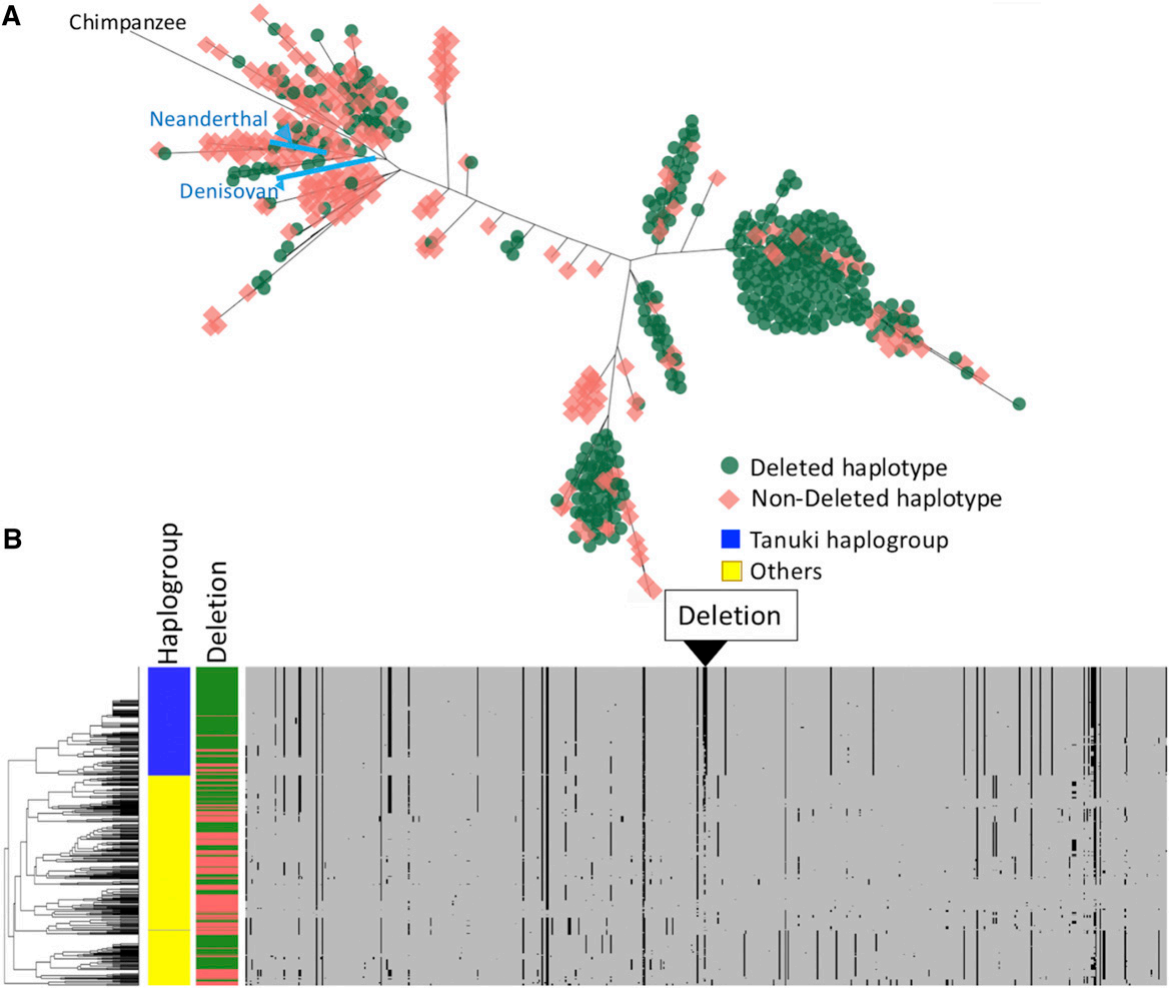
Haplotypes of the combined target1 and target2 regions. (A) The phylogenetic relationship of the downstream target 2 region of CEU, YRI, CHB, Neanderthal, Denisovan and Chimpanzee constructed by the maximum-likelihood method. The *GSTM1*-deleted haplotypes were marked by green and the *GSTM1*-non-deleted haplotypes were marked by red manually. (B) Clustered haplotypes of the upstream target 1 region and downstream target 2 regions of CHB and CEU. The clustering was conducted based only on the haplotypes themselves with neither *a priori* input of haplogroups nor the deletion status. The *GSTM1*-deleted haplotypes were marked by green and the *GSTM1*-non-deleted haplotypes were marked by red. The *Tanuki* haplogroup, which showed high population differentiation, was marked by blue and other haplotypes were marked by yellow.

Vcftools (Danecek *et al.* 2011) was used to calculate R^2^ values between the *GSTM1* deletion and flanking single nucleotide variations in Han Chinese in Bejing, China (CHB), Utah residents with Northern and Western European ancestry (CEU) and Yoruba in Ibadan, Nigeria (YRI) populations in 1KGP phase 3 datasets (Figure 2A, Figure S2). The Ensembl genome browser (http://asia.ensembl.org/index.html) was used to calculate and visualize linkage disequilibrium (LD) between single nucleotide variations and that (or LD) between the deletion and single nucleotide variations. Based on the R^2^ values, we used two target regions for the statistical neutrality tests. Two target regions were selected for the following analyses; target1, upstream the deletion (chr1:110211681-110223007 in the hg19) and target2, downstream the deletion (chr1:110246810-110255596 in the hg19), which showed relatively high R^2^ with the *GSTM1* deletion in CHB (Figure 2A). We constructed a phylogenetic tree of the combination of these two regions by VCFtoTree (Xu *et al.* 2017a) and visualized it in Dendroscope (Huson *et al.* 2007). For the clustering and the visualization of haplotypes and its cluster analysis, we used the pipeline described in Xu *et al.*, (2017b). The clustering was conducted based only on the haplotypes themselves with neither *a priori* input of haplogroups nor the deletion status (Figure 3).

#### Tests of natural selection on the Target region:

We first constructed a null distribution by calculating the *F_ST_* (Weir and Cockerham 1984), Tajima’s D (Tajima 1989), π (Nei and Li 1979) and iHS (Voight *et al.* 2006) values for 1000 randomly chosen regions on the same chromosome where the *GSTM1* is located (chromosome 1) and also match our target region by size (∼9kb). Then, we calculated these statistics observed in the downstream regions of the *GSTM1* deletion (chr1:110246810-110255596, downstream the deletion, also represented in Figure 2) and compare the results with those from the random regions. We also replicated this with size-matched regions that overlap with all 618 segmental duplications on chromosome 1 to match the genomic features in the *GSTM1* region (Figure S3).

We used genomic data of CEU, CHB and YRI populations in 1KGP. Tajima’s D value, iHS and π were calculated for 3kb intervals. XP-CLR were calculated for 2kb intervals. All these measurements were acquired through the 1000 Genomes Selection Browser (Pybus *et al.* 2014).

To visualize the geographic distribution of the *Tanuki* haplogroup, which was linked with the deletion in East Asia, we used data from 15 populations in the 1000 Genome Project for haplotype distribution analysis: BEB, CDX, CHB, ESN, FIN, GBR, GWD, IBS, JPT, KHV, LWK, MSL, PJL, TSI, and YRI, which have not experienced recent population admixture or migration. We used the “rworldmap” package (South 2011) to visualize the global distribution of the haplotypes.

#### Simulations for *F_ST_* and π values:

For the simulation analyses, we used ms (Hudson 2002) to conduct 1000 simulations matching the size (8,787bp) and the observed Watterson Estimator (θ=9.14) which was calculated by DnaSP (Librado and Rozas 2009) of the downstream target2 region (Figure 2). For each simulation, we generated 216 and 206 haplotypic sequences for YRI and CHB, respectively. We chose these populations and the number of sequences to match the empirical data from the 1000 Genome Project. To accurately model the effect of the demography in our simulations, we used the published parameters for East Asian and African populations (Schaffner *et al.* 2005) and we implemented the recombination rates of this region (ρ=5) reported by the HapMap Consortium (International HapMap Consortium *et al.* 2007). Once the sequences are generated, we focused on constructing expected distributions for population differentiation (*F_ST_*) and nucleotide diversity (π).

The command line for ms simulator was ./ms 422 1000 -t 91.4 -I 2 216 206 -m 1 2 3.2 -n 2 0.077 -en 0.0005 1 0.25 -en 0.001 2 0.077 -ej 0.00875 2 1 -en 0.0425 1 0.125 -r 5 8787.

We calculated *F_ST_* values between YRI and CHB populations for each single nucleotide variant that was generated in the simulations. We then plotted the values with the matching allele frequencies (0.69-0.70, the range of standard deviation of the observed allele frequency of the variations in *Tanuki* haplogroup) for each of these variants. Next, we considered the distribution of the simulated π values. To simulate this, we first generated 1000 simulated datasets as described above. We then focused on the segregating sites with alternative allele frequencies at 0.69-0.70, the range of standard deviation of the observed allele frequency of the variations in *Tanuki* haplogroup. Based on this, we calculated π for each of these haplogroups which carry the alternative allele for each single nucleotide variation in the simulated CHB population (Figure. S4).

We observed that the correlation fit between the simulated and empirical *F_ST_* values are high (Figure 5A), but notably less than the correlation between the simulated and empirical π values (Figure 5B). We believe that this is due to the fact that we used demographic parameters for two populations (East Asian and African populations (Schaffner *et al.* 2005) for the *F_ST_* simulations, while assumed parameters for only the East Asian populations for the π calculations.

#### The Run of Homozygosity analysis:

We used the demographic model of (Schaffner *et al.* 2005) with theta = 0.00375 per site and Rec rate = 0.001 per site and 4*Ne*m = 9 (*Ne* = effective population size *and* m= migration rate) from the empirical observation. We ran more than 7000 simulations per each condition and obtained 70-120 core sites with the frequency of 0.69+-0.031 (Standard deviation calculated from Binomial Distribution) in East Asia and 0 in Africa under neutrality. We calculated both side of ROH for the 70-120 core sites from the simulation. We also calculated one side (downstream) of ROH of the 238 *Tanuki* homozygous individuals in East Asian populations for the *Tanuki* SNP as there was a recombination hotspot in the upstream of *Tanuki* region (Figure. 2B). We used three recombination rates (20, 200, 2000 recombinations per the 200kb region) to see the impact of recombination rates on ROH. r = 200 was the most realistic parameter from the observation.

The command line for ms simulator was ./ms 422 1000 -t 750 -r 200 200000 -I 2 206 216 9 -en 0.0005 2 0.25 -en 0.001 1 0.077 -en 0.0425 2 0.125

To test for potential sampling bias (*e.g.*, consanguinity) in our dataset, we conducted bootstrap resampling of individual phased chromosomes. There are 238 individuals in East Asian populations (CHB, JPT, CHS, KHV, CDX) that are homozygous for *Tanuki* haplotype. To construct a distribution to investigate whether there is a sampling bias, we randomly resampled from 694 phased *Tanuki* haplotypes to generate 500 datasets each with 238 homozygous constructs. This allowed us to test whether the observed value of ROH deviates from randomly resampled distribution. We observed no such deviation, suggesting no sampling bias in our analysis (Figure S5).

#### Age estimation of *Tanuki* haplogroup and selection on *Tanuki* haplogroup:

We conducted several age estimates in this paper. First, we used the simple formula detailed (15) in (Kimura and Ohta 1973) and estimated the expected time for the *Tanuki* haplogroup to reach the current frequency in East Asia and Europe under neutrality.

Second, we used the average sequence differences of the *Tanuki* haplogroup from a non-*Tanuki* haplotype (Figure S6, S7) to calculate a coalescent date for the divergence of these two haplogroups. Specifically, based on a more recent (5 million years (Varki and Altheide 2005)) and older (∼12 million years (Moorjani *et al.* 2016)) divergent estimates between humans and chimpanzees, we estimated the coalescent of *Tanuki* haplogroup (0.001048 distance from the root haplotype) to be between 300 to 720 thousand years before present.

Third, we calculated the starting date of the putative selection on *Tanuki* haplogroup based on the run-of-homozygosity in this locus with method detailed by Racimo *et al.* (2015). Briefly, at a past time when r*L*t (r = recombination rate, L = the length of run of homozygosity, t = the time when the selection on the variant) reaches 1, the ROH shows rapid breaks because of recombination. So, we can assume that the t at that time is the start point of the selection. We used r= (1.45±0.05)× 10^−8^/generation (Narasimhan *et al.* 2017). In the paper, they noted this value and confidence interval from their two estimates for the mutation rate (1.51 and 1.41 × 10^−8^ per bp per generation (Narasimhan *et al.* 2017), u = 3 × 10^−8^/site/generation, and observed averaged ROH_aver_ of the *Tanuki* Haplogroup (∼16kb).

It is important to note some caveats here. First, the coalescence dates depend on the local recombination and mutation rates, and even our best estimates may not be accurate with our current knowledge. Second, given that this locus has likely evolved under adaptive forces, it would be more appropriate to incorporate dating approaches that consider selection. However, we have little insight into the timing and the strength of the selection, and thus we focused on dating approaches that do not depend on selection coefficient as a parameter.

## Data availability

Table S1 contains genotyping of the *GSTM1* copy number by ddPCR of 17 individuals in YRI population in the 1KGP. Supplemental materials are available at Figshare: Saitou, Marie; Gokcumen, Omer; Satta, Yoko (2018): G3-Supp0626.pdf.; https://doi.org/10.25387/g3.6384782.

## Results

### Imperfect linkage disequilibrium Between the *GSTM1* deletion and flanking single nucleotide variants in the human lineage

To understand the evolutionary mechanisms which maintain the *GSTM1* deletion common in humans, we first attempted to resolve the haplotype structure of the locus. We calculated the R^2^ value between the *GSTM1* deletion and flanking single nucleotide variants in Han Chinese in Bejing, China (CHB), Utah residents with Northern and Western European ancestry (CEU) and Yoruba in Ibadan, Nigeria (YRI) populations in 1KGP phase 3 datasets (Figure 2A, Figure S1). Remarkably, we found almost no linkage disequilibrium between the *GSTM1* deletion flanking single nucleotide variants in CEU and YRI (Figure S1), and observable, but imperfect linkage disequilibrium in CHB only extending to >2.5kb on each side of the deletion (R^2^=∼0.45,Figure 2A). One way to explain this low level of linkage disequilibrium is by invoking a recombination hotspot in the region. However, we found that the reported recombination in both deCODE (Kong *et al.* 2010) and 1000 genomes phase 3 datasets for the region (1000 Genomes Project Consortium *et al.* 2010) is high, but not exceptionally high to be categorized as a hotspot (3-17 cM/Mb, (Mc Vean *et al.* 2004)) (Figure 2B).

One mechanism that can explain the imperfect linkage disequilibrium between the deletion and the neighboring variants can be recurrent formation of deletions. In other words, if two or more independent deletion events have occurred in different haplotypic backgrounds, then we expect (i) different deletion breakpoints, and (ii) LD to be intact between the variants on each side of the deletion as described by Boettger *et al.* (Boettger *et al.* 2016). In a previous study, we conducted an analysis of the breakpoints of this deletion, but, due to repetitive nature of this locus, could not identify the exact breakpoints of this deletion (Saitou *et al.* 2018). In parallel, we found that there is relatively weak, but observable linkage disequilibrium (R^2^=∼0.5) between variants on each side of the deletion in CHB (Figure 2C). Overall, our results cannot rule out recurrent formation of the *GSTM1* deletion in the human lineage.

To further understand the haplotypic variation in this locus, we constructed a phylogenetic tree using sequence variation data in the “target” region flanking the deletion for 620 phased haplotypes from YRI, CEU, CHB populations, as well as chimpanzee, Denisovan, Neanderthal genomes (Figure 3A). As expected from the low levels of linkage disequilibrium between single nucleotide variants and the deletion, we observed no clear separation between haplotypes with and without the deletion. Instead, we found multiple branches that are predominantly populated with deleted haplotypes and others with non-deleted haplotypes without notable population structuring. These observations are concordant with the recent study documenting the genetic variation in this locus in a Russian population (Khrunin *et al.* 2016). It is important to note here, however, that Neanderthal and Denisovan haplotypes, as well as the branching point for the chimpanzee reference haplotype, all cluster with the predominantly non-deleted haplotypes. This suggests that the deletion is a derived variant that likely evolved after the human-Neanderthal divergence (*i.e.*, in the last 1 Million years).

Despite these general insights, the observable lack of linkage disequilibrium between the *GSTM1* deletion and the neighboring variants reduces the power of disease-association studies (*e.g.*, GWAS) that rely on imputation of the *GSTM1* deletion genotype using nearby single nucleotide variants. Even considering multiple single nucleotide variants within a single population, we were not able to impute the deletion accurately in any of the study populations (YRI, CHB, and CEU) (Figure 3B). This is concordant with the previous finding reported the difficulty to predict the copy number of the *GSTM1* gene by the flanking haplotypes in CEU (Khrunin *et al.* 2016). As such, direct genotyping, rather than imputation, may be more robust approach to study *GSTM1* deletion.

### A potential signature of an incomplete sweep in East Asian populations in the *GSTM1* locus

**Figure 4:**
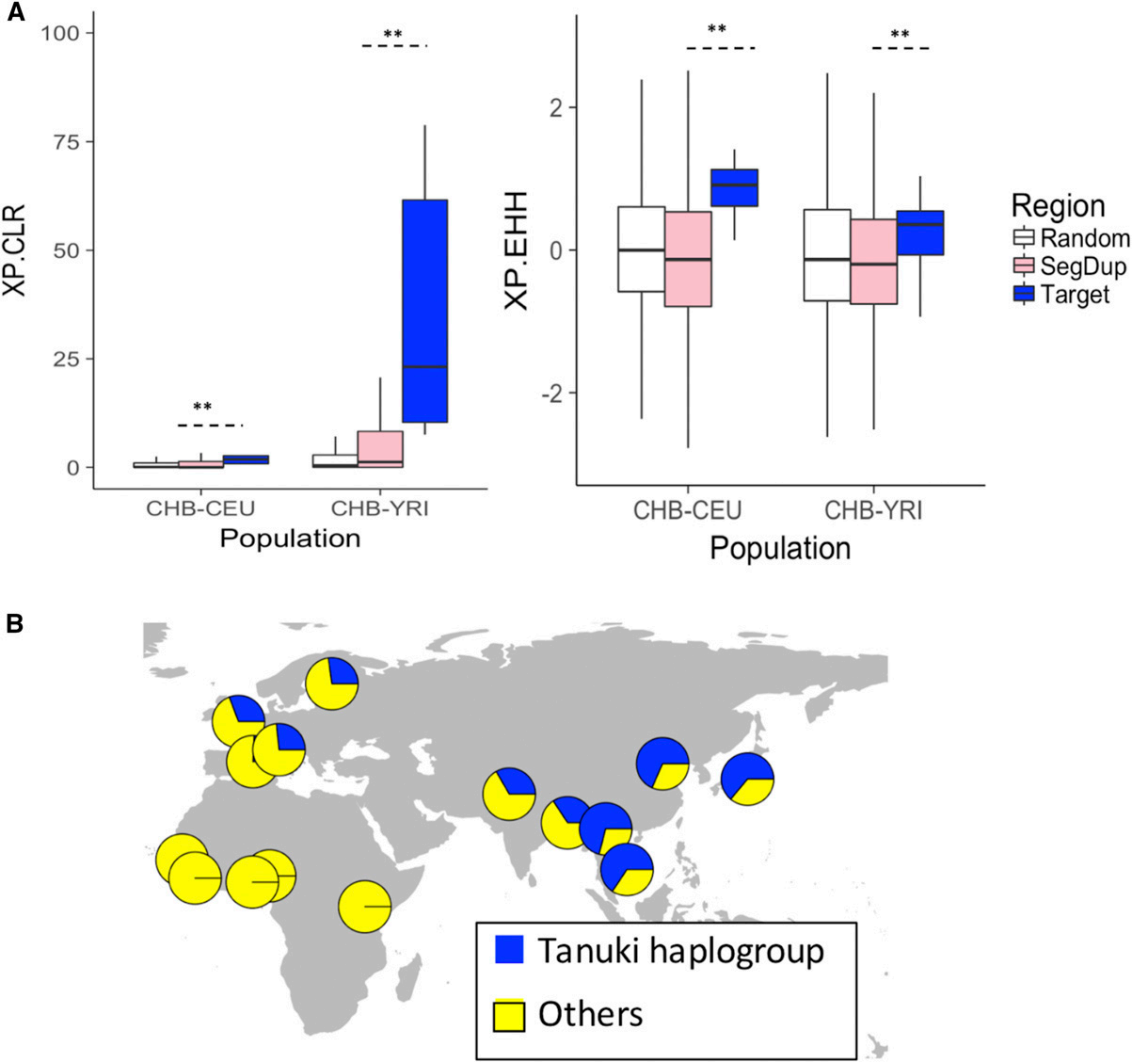
(A) Neutrality tests on the target region (chr1:110246810-110255596, downstream the deletion, also represented in Figure. 2.), the 1000 randomly selected 9kb regions, and all the segmental duplications on chromosome one, which contains the *GSTM1* in CEU, CHB and YRI populations in 1KGP. XP-CLR and XP-EHH were calculated for 2kb intervals. The stars show significant differences (*P* < 0.01, Wilcoxon Rank Sum Test). (B) The geographical distribution of the *Tanuki* haplogroup calculated from the 1KGP phase3 data.

To reveal any potential signatures of adaptive evolution affecting variation in this locus, including the *GSTM1* deletion, we conducted statistical neutrality tests using single nucleotide variation data from the sequences flanking the *GSTM1* deletion. Specifically, we first constructed a null empirical distribution by calculating the *F_ST_* (Weir and Cockerham 1984), Tajima’s D (Tajima 1989), π (Nei and Li 1979), iHS (Voight *et al.* 2006), XP-EHH and XP-CLR values for 1000 randomly chosen regions on the same chromosome where the *GSTM1* is located (chromosome 1) and also match our target region by size (∼9kb). Then, we calculated these statistics observed in the downstream regions of the *GSTM1* deletion and compared the results with those from the random regions (Figure 4A, Figure. S2). We also replicated this with randomly selected, size-matched regions that overlap with all of the 618 segmental duplications on chromosome 1 to match the genomic features in the *GSTM1* region (Figure. S2). We found that both XP-CLR and XP-EHH tests showed significant differences as compared to neutrality when *CHB* population is involved (*p* values < 0.01, Wilcoxon rank sum test with continuity correction). These tests measures the change in allele frequency in one population has occurred more quickly than expected by drift alone and the difference in extended homozygosity between two populations (Figure 4A, Table 1). XP-EHH (Sabeti *et al.* 2007) is cross-population extended haplotype homozygosity and XP-CLR (Chen *et al.* 2010) is multi-locus allele frequency differentiation between two populations. Collectively, the unusually high XP-CLR and XP-EHH values in CHB population suggest an incomplete sweep has shaped the distribution of *Tanuki* haplogroup in this population.

**Table 1:** XP-EHH and XP-CLR between CHB-YRI, CEU-YRI and CHB-CEU. Significant values were shaded.

XP-CLR			
XP-EHH	YRI	CEU	CHB
YRI		3.18 (*P* = 0.244)	35.6 (*p*=0.000707)
CEU	−0.338 (*P* = 0.995)		3.54 (*p*=0.00543)
CHB	0.232 (*p*=5.32e-0.7)	0.902 (*p*=2.20e-16)	

To investigate the haplotypic background of this putative sweep, we focused on the single nucleotide variants that showed the highest population differentiation (Figure 4A). We first showed that they are in high linkage disequilibrium with each other (R^2^ > 0.84), indicating that they represent a single haplotype group in East Asian populations (Figure S8, Table S2, called *Tanuki* haplogroup from hereon) in the target2 region (Figure 2A). We first investigated the global distribution of the haplogroup and found that it represents the major allele (>70% allele frequency) in East Asian populations, but it is virtually absent in sub-Saharan African populations (Figure 4B, Figure S9).

To determine the age of this haplogroup, we constructed a phylogenetic tree (Figure S6) of all *Tanuki* haplotypes in CEU and CHB populations (no *Tanuki* haplotype was found among YRI genomes), along with a non-*Tanuki* haplotype and the chimpanzee reference genome haplotype as outgroups. We surmised that *Tanuki* haplotype diverged from other human haplotypes at least 300 thousand years ago (∼300K to 720K YBP). Even considering the relatively large deviation of this estimate, this date is far earlier than the out-of-Africa migrations. As such, we conclude that this haplogroup has originated in Africa, but has increased in frequency in Eurasia. It is plausible that some *Tanuki* haplotypes still remain in African populations, but at a low allele frequency.

To further understand the haplotypic background of the significantly high XP-CLR and XP-EHH values in Asian populations as compared to European and African populations, we constructed a network of *Tanuki* haplotypes (Figure S7). We found that a vast majority of *Tanuki* haplotypes in CHB population has identical sequences, suggesting a rapid and recent increase in the allele frequency in the East Asian populations. This observation explains the unusually high XP-CLR and XP-EHH values observed for CHB population and further support the notion that a incomplete sweep has shaped the distribution of *Tanuki* haplogroup in this population.

### Allele frequency of the *Tanuki* haplogroup is unusual in East Asia, but not in Europe

The high XP-CLR and XP-EHH values along with very high frequency of *Tanuki* haplogroup in CHB potentially point to the effect of an local sweep. The alternative, null hypothesis would be that the observed difference in allele frequency is due to the effect of drift alone. To further distinguish between these two scenarios, we conducted multiple empirical and simulation based analyses. First, we investigated whether the allele frequency of *Tanuki* haplogroup in East Asian and European populations can be explained by drift alone. To do this, we first assumed that the putative target for selection is an allele that reached to approximately 70% allele frequency in East Asia, but remain at 25% allele frequency in Europe (Figure 4B). Then, we used the simple formula detailed (15) in (Kimura and Ohta 1973) and estimated that it would take ∼580 and ∼160 thousand years under neutrality for that single haplotype to increase in allele frequency from 1/2*N_e_* in ancestral African population to 70% and 25% observed in Asia and Europe, respectively. This results suggest that the allele frequency of *Tanuki* haplotypes in Europe can be explained by neutrality alone, but not in East Asia.

To further interrogate whether the high allele frequency of *Tanuki* haplotypes can be explained by genetic drift alone, we conducted simulation-based analyses of the genetic variation we observed (for detailed conditions for simulations, see Methods). Specifically, we used ms (Hudson 2002) to generate 1000 simulated datasets comprising sequences matching the size (8,787bp) of the target2 region of *GSTM1* (Figure 2). For these simulations, we used demographic parameters previously laid out for CHB and YRI populations (Schaffner *et al.* 2005) to accurately model the effect of drift on nucleotide diversity and allele frequency.

**Figure 5:**
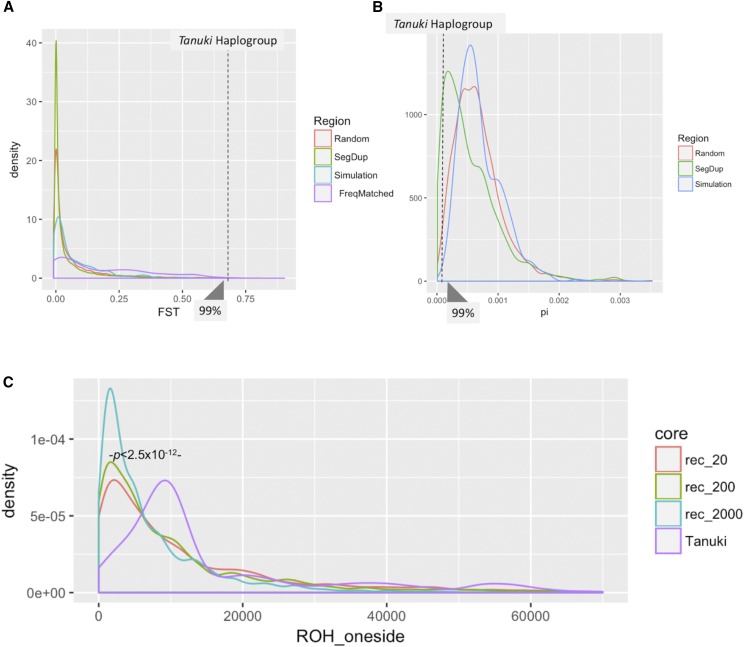
(A) The average values of *F_ST_* for *Tanuki* SNPs (dotted line), the 1000 randomly selected 9kb regions (red), 9kb regions on chromosome 1 that overlap with segmental duplications (green), simulated data under neutrality (blue), and frequency-matched (0.69 - 0.70) single nucleotide variants on chromosome 1 (purple). We found that none of 603 frequency-matched simulated *F_ST_* is higher than the observed *F_ST_* for the *Tanuki* haplogroup. (B) The π value for *Tanuki* haplotypes (dotted line), the 1000 randomly selected 9kb regions (red), 9kb regions on chromosome 1 that overlap with segmental duplications (green), simulated data under neutrality (blue). The results showed that π observed in the *Tanuki* haplogroup is lowest among 389 frequency-matched simulated values. (C) The Run of Homozygosity of the *Tanuki* SNP (purple) and simulated frequency-matched SNPs with 3 different recombination rates, 20 (red), 200 (green), 2000 (blue). We found that the ROH values observed for the *Tanuki* single nucleotide variants in the 1KGP East Asian populations is significantly higher than what is expected under neutrality based on frequency matched simulation results with r = 200, the most realistic parameter from the observation (Fig. 5C, *P* < 2.5x10^−12^, Wilcoxon-Rank-Sum Test).

Once the sequences were generated, we calculated *F_ST_* values for each single nucleotide variant that was generated in the simulations between two simulated populations (YRI and CHB). To verify the accuracy of our simulations, we first compared our simulated sequences to empirical distribution of *F_ST_* between YRI and CHB populations for randomly selected single nucleotide variants across the genome (Figure 5A). We then plotted the *F_ST_* values with the matching allele frequencies (0.69-0.70, that represent the deviation of the observed allele frequency of the variations in *Tanuki* in Asian populations) for each of these variants observed in the simulation. We found that none of 603 frequency-matched simulated *F_ST_* is higher than the observed *F_ST_* for the *Tanuki* haplogroup (**>99^th^ percentile**, Figure 5A). Overall, these results suggest that the unusually high allele frequency of *Tanuki* haplotypes in CHB population is unlikely to be explained by neutral forces alone.

### Haplotype homozygosity provides further evidence for a selective sweep

In parallel to the allele frequency based analyses described above, we conducted tests based on haplotype similarity. We first followed the reasoning outlined by Kim and Satta (Kim and Satta 2008). Briefly, if a recent sweep indeed increased the allele frequency of a particular existing haplotype group, we expect that the nucleotide diversity would decrease among the haplotypes that were sweeped, but not the others. Indeed, we found that the nucleotide diversity of haplotypes that belongs to the *Tanuki* haplogroup, which make up >70% of haplotypes in East Asians, is approximately six times lower (π=0.00012) in East Asians as compared to the nucleotide diversity observed in haplotypes that do not belong to the *Tanuki* haplogroup in the same population (π=0.00076).

To explore this issue, we have considered the π values for *Tanuki* and *nonTanuki* haplotypes across a larger genomic region surrounding the *GSTM1*, including sequences that are not overlapping segmental duplications in multiple East Asian populations (CHB, CHS, JPT and KHV) (Figure. S10). These results also corroborates our initial finding that *Tanuki* haplotypes show unusually low π as compared to simulated expectations (Figure. 5B).

To quantify this observation, we used simulated sequences (as described above) to construct an expected, neutral distribution of nucleotide diversity (π) values. Specifically, we wanted to test whether π calculated for the haplotypes that belong to the *Tanuki* haplogroup is lower than expected distribution under neutrality. To simulate this, we first generated 1000 simulated datasets as described above. From the simulated dataset, we chose haplogroups within the simulated with alternative allele frequencies at 0.69-0.70 to match that of *Tanuki* haplogroup in frequency (please see Figure S4). Then, we plotted the π values for these simulated haplogroups. The results showed that π observed in the *Tanuki* haplogroup is lowest among 389 frequency-matched simulated values (**<1^st^ percentile,** Figure 5B).

To test whether the low nucleotide diversity observed in *Tanuki* haplogroup is also translated into extended homozygosity, a hallmark of a selective sweep, we simulated expected Runs of Homozygosity (ROH) assuming demographic parameters outlined in (Schaffner *et al.* 2005) and different recombination rates (see methods for details). We found that the ROH values observed for the *Tanuki* single nucleotide variants in the 1KGP East Asian populations is significantly higher than what is expected under neutrality based on frequency matched simulation results with r = 200, the most realistic parameter from the observation (Figure 5C, *P* < 2.5x10^−12^, Wilcoxon-Rank-Sum Test). The averaged ROH of the *Tanuki* haplogroup was 16Kb.

**Figure 6:**
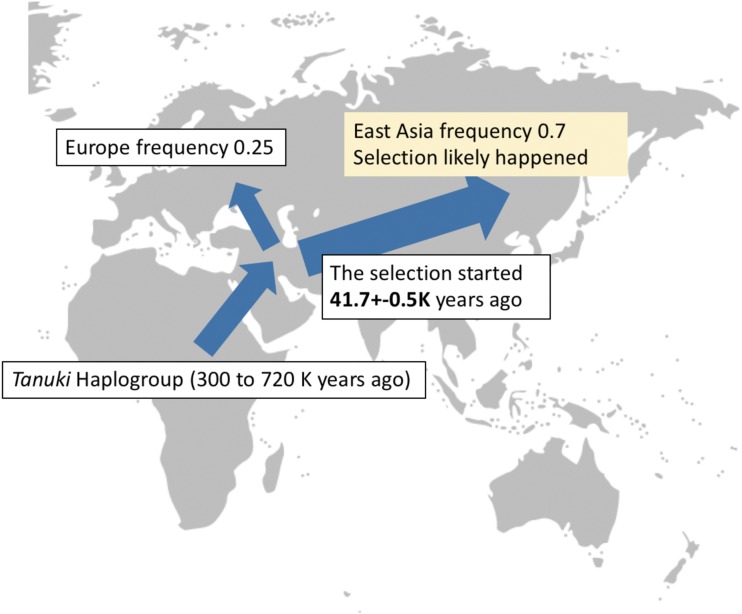
The evolutionary history of the *Tanuki* haplogroup. Based on our analyses, we argue that the *Tanuki* haplogroup originated before Out of Africa migrations (∼300K years before present) and spread out to Eurasia. We further argue that in Europe, the frequency of Tanuki haplogroup increased approximately to 0.25 under neutrality. In Asia, the selection on the *Tanuki* haplogroup started 41.7K years ago and pushed frequency of the *Tanuki* haplogroup to 0.7 under selection.

This calculation also allowed us to calculate a potential date for the sweep using the approach outlined elsewhere (Racimo *et al.* 2015) (see methods). Briefly, this method uses the length of ROH to estimate the age of the beginning of a sweep under given recombination and mutation rates. Using this method, we estimated this date to be 41.7±0.5K years ago when the selection on *Tanuki* Haplogroup began (Figure 6). Combined, the observed values of population differentiation and haplotype diversity are inconsistent with the neutral evolution of this locus and are parsimonious with our hypothesis that the *Tanuki* haplogroup has increased its frequency under adaptive evolution in the East Asian populations.

### Potential functional impact of *Tanuki* haplogroup

By carefully resolving the different haplotype groups in the *GSTM1* locus, we were able to detect a putatively adaptive haplogroup (*Tanuki* haplogroup). The exact underlying evolutionary reason and the functional impact of the increase in frequency of the *Tanuki* haplogroup remain excellent venues for future studies. One obvious question is whether the *GSTM1* deletion is the actual target of positive selection. Indeed, we discovered that *Tanuki* haplogroup is significantly, albeit imperfectly, linked with the *GSTM1* deletion in the CHB population (R^2^=∼0.47) (Table S2). It is important to note that despite the low linkage disequilibrium, ∼91% of the *Tanuki* haplotypes harbor the *GSTM1* deletion. We reasoned that if the *GSTM1* deletion was the target for the selective sweep that we observed, then other haplotypes carrying the *GSTM1* deletion also show evidence for selection. However, we did not observe any unusual increase in allele frequency of other haplotypes that carry the deletion. As such, it is highly plausible, as drastic an event as a whole gene deletion is, *GSTM1* deletion may not be the main target of the selective sweep that we observed.

To further follow up this thread, we asked whether the *GSTM1* gene has no fitness consequences and hence accumulating loss of function mutations, such as the deletion. Under this scenario, the *GSTM1* deletion just swept along with the remainder of the *Tanuki* haplogroup with scant adaptive consequences. Contradicting this possibility, it was reported that the *GSTM1* gene sequence is conserved among great apes, as well as between humans and archaic humans. Specifically, we found only 4 nonsynomous variants between human and chimpanzee *GSTM1*, and found none among human, Neanderthal and Denisovan *GSTM1* genes (Table S3). In addition, only two commonly observed nonsynonymous mutations were reported for *GSTM1* gene in human population, both of which have little effect on the *GSTM1* function (Moyer *et al.* 2007; Tatewaki *et al.* 2009). ExAC database allowed us to be more specific (Lek *et al.* 2016). The expected number of loss-of-function variants at the *GSTM1* locus is 8.4 but observed number was 2 in the Exome analysis in 60,706 humans. In addition, the frequency of these loss-of-function variants are extremely low (<0.00003133). It is important to note that the deletion of the entire gene in approximately half of human genomes is not currently reported in ExAC database. However, our point here is that if *GSTM1* has no fitness effect in humans, we expect to find relatively high frequencies of loss of function variants in addition to the deletion. Instead, virtually all the nondeleted *GSTM1* haplotypes carry an intact, open-reading frame, further supporting the notion that both deleted and nondeleted haplotypes are maintained in the population.

These results suggest that it is likely that the *GSTM1* deletion has fitness effects, but it is independent of the selective sweep we observed for the *Tanuki* haplogroup. To explain these observations, we hypothesize that *Tanuki* haplogroup affect the function of multiple genes in the *GSTM* gene family and not just *GSTM1*. Consequently, the putative adaptive phenotype is a result of combination of these effects. Such a perspective has precedent where other studies described likely adaptive haplotypes that have effects on multiple metabolism gene families, such as *IRX* (Claussnitzer *et al.* 2015) and *FAD* (Fumagalli *et al.* 2015).

To test whether *Tanuki* haplogroup indeed affect other neighboring genes, we investigated functional variants that are linked with this haplogroup. Specifically, we identified variants with unusually high PHRED scores (>90^th^ percentile) (Kircher *et al.* 2014), GWAS variants (MacArthur *et al.* 2017), and nonsynomous variants (Figure S11). We found no nonsynomous or GWAS variants that is linked with the Tanuki haplogroup. Nevertheless, we found one variant that has a high PHrED score (rs61799140). It is thus plausible that *Tanuki* haplogroup may have regulatory effect on nearby genes. To further test this, we used gene expression data from Gtex portal (Lonsdale *et al.* 2013; Wheeler *et al.* 2016). Our results showed that this haplogroup is associated with significant decreases in expression of the *GSTM5* gene in most tissues, especially in the brain, but increases the expression of *GSTM3* in skeletal muscle (Figure S12). As such, one potential argument would be that the selected effect is not the functioning of a single gene, but the overall regulatory impact of the *Tanuki* haplogroup on the *GSTM* locus.

## Discussion

### Working With *complex* genomic structural variation loci

Our results provide a case study for the evolutionary impact of common haplotypic variation in a complex locus, involving both functional single nucleotide and copy number variants. One particular challenge in this locus that we verified is the general lack of linkage disequilibrium between common, neighboring variants. Specifically, we found a lack of strong linkage disequilibrium between the *GSTM1* deletion and neighboring variants. This finding corroborates our previous work at a deeper evolutionary depth, where we found evidence for multiple gene conversion events differentiating human and chimpanzee *GSTM* locus (Saitou *et al.* 2018). As such, most genome-wide association studies would not have effectively interrogated the potential biomedical impact of the *GSTM1* deletion because they have only investigated the single nucleotide variants, which do not adequately tag the deletion variant. In fact, when we consider the best case scenario for such studies and focus on East Asia, where the linkage disequilibrium is relatively high (R^2^ = 0.4), statistical power for a single nucleotide variant based genome-wide association study would still be low. For example, based on Hong and Park (2012)’s calculations, the statistical power to detect an association would be only 26.5% when assuming a relatively standard experimental setup (*e.g.*, the odds ratio of the phenotype 1.3, 1000 cases and 1000 controls, *etc*.).

This is not an exceptional case since the majority of deletions reside in similar haplotypic architectures (Sudmant *et al.* 2015b), complicating both evolutionary analysis, as well as disease association studies that depend on imputation to interrogate structural variants. Almost one-third of the deletions reported in 1000 genomes have R^2^ < 0.6 with neighboring variants (Sudmant *et al.* 2015b). As such genome-wide association studies will lose significant amount of power when assessing the effect of such SVs on tested trait if they are using imputation-based genotyping. Consequently, it is important to conduct direct genotyping of the deletion for such analyses or carefully resolve the haplotype structure within the locus to accurately assess the biomedical impact of these variants. Such locus-specific analyses indeed led to important connections between several structural variants and human diseases (Rothman *et al.* 2010; Usher *et al.* 2015; Boettger *et al.* 2016).

### Evolution of the *GSTM1* locus

Here, we report an incomplete sweep that increase the allele frequency increase of n existing haplogroup (*Tanuki*) in East Asian populations, putatively affecting the function of multiple *GSTM* genes (as summarized in Figure 6). Recent studies have shown the importance of sweeps on standing variation (Schrider and Kern 2017), rather than hard sweeps on novel variants (Hernandez *et al.* 2011), to be the dominating type of positive selection acting on human genome.

These insights fit well with our observations for the *Tanuki* haplogroup as well as with the emerging, broader picture where genes involved in metabolism have been shown to evolve under complex adaptive forces. For example, the *GSTM1* deletion and multiple *NAT* (N-acetyltransferase) variants were often considered together as the leading candidates for explaining the genetic basis of bladder cancer susceptibility (García-Closas *et al.* 2005). *NAT2* has been reported to be evolving under balancing selection (Mortensen *et al.* 2011). In addition, variations in other xenobiotic metabolism genes, such as members of the *SLC* (solute carrier) gene family, have also been shown to be evolving adaptively (Sabeti *et al.* 2007). Some of these variations involve gene deletions. For example, the deletion of *UGT2B17* (uridine diphosphoglucuronosyl-transferase) has been shown to have increased in frequency in East Asian populations under adaptive forces (Xue *et al.* 2008). Overall, functional variation affecting metabolizing genes may be maintained adaptively in the human populations due to varying environmental pressures.

### Implications to evolutionary medicine

As mentioned above, the variation in the *GSTM* locus has been the subject of more than 500 studies within the context of multiple diseases (Parl 2005). However, given that majority of these studies are correlations, they lack mechanistic or evolutionary insights as to why the genetic variation in this locus is relevant. The emerging picture is that of a gene family (*GSTM1-5*) that broadly metabolizes multiple carcinogenic substances, for example, 4-nitroquinoline-1-oxide (NQO) (Hayes *et al.* 2005). On top of that functional layer, it has been shown that this gene family is riddled with common genetic variation, including the unusually common deletion of the *GSTM1*. These genetic variants, as expected, were associated with multiple cancers (Parl 2005). The mechanistic explanation would be that the reduced the *GSTM* function leads to higher susceptibility to carcinogenic substance exposure (Hayes *et al.* 2005).

However, there seems to be some functional redundancy among the *GSTM* gene family members. As a consequence, the association between single variants to traits may not completely capture the biomedical impact of the variation in this locus as a whole. For example, the *GSTM2* may compensate some of the lost function due to the *GSTM1* deletion (Bhattacharjee *et al.* 2013). As such, the overall functional impact of a variant depends on its genomic context. Moreover, a recent pathway-level analysis revealed that glutathione conjugation pathway, for which the *GSTM* genes are central, is a significant player in determining breast cancer susceptibility (Wang *et al.* 2017). The implication being that rather than single variants, the combined effect of multiple variants affecting the function of the genes in this pathway eventually contributes to the overall disease susceptibility. This is not a new concept (Jin *et al.* 2014). Such insights into the collective but varied impact of variation in a given locus have been discussed in evolutionary context in multiple species (Lin *et al.* 2015; Salojärvi *et al.* 2017). We are now in a position to quantitatively address this issue, especially using genealogical approaches as we described for the *GSTM1* here.

From an evolutionary medicine point of view, it is important to highlight two interrelated features of the *GSTM* locus. First, even when a single haplotype affects susceptibility to disease, this may be due to pleiotropic effects. Indeed, we described multiple putative functional effects of the *Tanuki* haplogroup in East Asian populations, including the loss of function due to the *GSTM1* deletion and the decrease of the *GSTM5* expression. This result exemplifies the benefits of a haplotype-level understanding of the genetic variation. We argue based on our results and those of others (Liu *et al.* 2005; Claussnitzer *et al.* 2015) that the co-occurrence of multiple functionally and biomedically relevant variants in particular haplotypes should be treated as the norm, rather than the exception. As exemplified in this study, functional analysis of haplotypes that are under selection may provide crucial targets for future mechanistic studies.

Second, our results add to the growing list of population-specific haplotypes that may contribute to disease susceptibility, further underlying the importance of conducting genetic epidemiology studies in ancestrally diverse populations (Rosenberg *et al.* 2010; Wojcik *et al.* 2017). This is especially pertinent to the *GSTM* locus, given that environmental toxins and carcinogens, which may vary from one population to the other, are the primary target for this gene family. Such loci are also targets for local selection in humans, may be best exemplified by the recent study on Arsenic adaptation in Argentinian Andes population (Schlebusch *et al.* 2015).

## Conclusion

In this study, we report a particular haplogroup (*Tanuki* haplogroup) carrying the deletion allele that has likely evolved under non-neutral conditions and reached a high allele frequency in East Asian populations. This haplogroup has a broad regulatory effect on the metabolizing *GSTM* gene family. Overall, our study adds to the emerging notion that complex loci involving and structural variants may contribute to adaptive and biomedically relevant phenotypic variation (Boettger *et al.* 2016; Sekar *et al.* 2016).
